# Ischemic Stroke as the First Manifestation of Takayasu Arteritis: A Case Report

**DOI:** 10.3390/neurolint18030057

**Published:** 2026-03-18

**Authors:** Dominika Jakubowicz-Lachowska, Magdalena Sarnowska, Monika Chorąży, Alina Kułakowska

**Affiliations:** Department of Neurology, Medical University of Bialystok, 15-276 Bialystok, Poland

**Keywords:** Takayasu arteritis, ischemic stroke, young adult, thrombolysis, mechanical thrombectomy, vasculitis reperfusion therapy, large-vessel occlusion

## Abstract

Introduction: Ischemic stroke in young adults is uncommon and is frequently associated with rare etiologies, including autoimmune diseases and vasculitis. Takayasu arteritis (TA) is a chronic inflammatory large-vessel arteriopathy involving the aorta and its major branches and may result in cerebral ischemia due to arterial stenosis or thrombosis. Case Presentation: We report the case of a 26-year-old woman with a history of suspected rheumatoid arthritis and Lyme disease who presented with acute left-sided hemiparesis and dysarthria. At admission, large-vessel vasculitis had not yet been suspected, and the patient was treated according to standard acute stroke protocols. Computed tomography angiography (CTA) revealed occlusion of the right middle cerebral artery bifurcation and the right common carotid artery, with inflammatory changes involving the brachiocephalic trunk and subclavian arteries. Intravenous thrombolysis (iv rtPA) was followed by mechanical thrombectomy (MT), resulting in neurological improvement. Outcome: Further diagnostic work-up confirmed TA, and immunosuppressive therapy with cyclophosphamide and infliximab was initiated. Conclusion: This case underscores the importance of considering inflammatory large-vessel disease in young patients presenting with acute ischemic stroke and illustrates that endovascular reperfusion may be feasible in this clinical setting.

## 1. Introduction

Ischemic stroke remains one of the leading causes of disability and death worldwide [1]. It predominantly affects older individuals with conventional risk factors such as hypertension, diabetes mellitus, and atrial fibrillation. However, approximately 10–15% of strokes occur in young adults and require extensive diagnostic evaluation to identify rare etiologies, including autoimmune and inflammatory vascular diseases [2].

Takayasu arteritis (TA) is a chronic idiopathic large-vessel vasculitis that affects major arteries such as the aorta and its primary branches, leading to vessel dilatation, narrowing, aneurysm formation, or occlusion [3]. Neurological manifestations, including ischemic stroke, may occur due to vascular stenosis, occlusion, or thrombosis [4,5]. Stroke is a recognized complication, reported in 10–20% of patients with TA. However, stroke as the first manifestation of TA is infrequent, and only a few cases have been reported [6]. We present a case of ischemic stroke as the first manifestation of TA, successfully treated with MT.

## 2. Case Presentation

A 26-year-old woman with a history of suspected rheumatoid arthritis and Lyme disease (currently receiving antibiotic therapy) was admitted to the Emergency Department with sudden onset of left-sided limb weakness and speech disturbance that developed upon awakening at 4:00 a.m. She was an active-duty soldier.

Her medical history included one full-term pregnancy, no history of miscarriages, regular menstrual cycles, a balanced diet, occasional alcohol consumption, and smoking approximately five cigarettes per day. On admission, she was afebrile, with normal blood pressure and heart rate, and oxygen saturation of 100% on room air. Physical examination of the chest and abdomen revealed no abnormalities. Initial laboratory tests were within normal limits. Neurological examination revealed left-sided hemiparesis and dysarthria. The initial National Institutes of Health Stroke Scale (NIHSS) score was 11, and the modified Rankin Scale (mRS) score prior to symptom onset was 0.

## 3. Diagnostic Assessment

**Figure 1:**
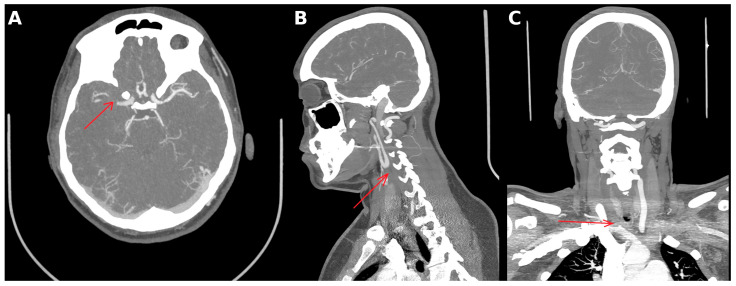
Computed tomography angiography (CTA). (**A**) Axial CTA demonstrated occlusion of the right middle cerebral artery bifurcation (arrow). (**B**) Sagittal CTA reconstruction showed complete occlusion of the right common carotid artery (arrow). (**C**) Coronal CTA reconstruction demonstrated occlusion at the level of the brachiocephalic trunk (arrow).

Computed tomography angiography (CTA) demonstrated occlusion of the right middle cerebral artery bifurcation and proximal M2 branches (Figure 1), as well as complete occlusion of the right common carotid artery filled with non-calcified thrombotic material. In addition, inflammatory wall changes were observed in the brachiocephalic trunk, right subclavian artery, right common carotid artery, and left subclavian artery. No acute ischemic lesions were detected on plain CT.

**Figure 2:**
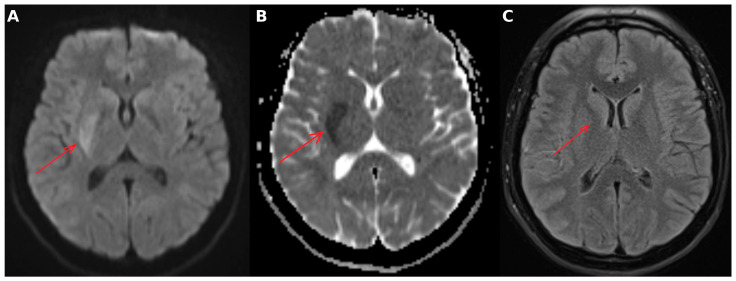
Brain magnetic resonance imaging (MRI). (**A**) Diffusion-weighted imaging (DWI), axial plane, demonstrated restricted diffusion in the right basal ganglia (arrow). (**B**) Apparent diffusion coefficient (ADC) map confirmed true restricted diffusion (arrow). (**C**) Fluid-attenuated inversion recovery (FLAIR) showed no corresponding hyperintensity, consistent with hyperacute ischemic infarction (arrow).

Subsequent MRI demonstrated a lesion with restricted diffusion, approximately 12 cm^3^ in volume, in the right basal ganglia, without corresponding hyperintensity on fluid-attenuated inversion recovery (FLAIR) sequences, consistent with hyperacute ischemic infarction (Figure 2).

### 3.1. Therapeutic Intervention

In the absence of contraindications and with no prior suspicion of large-vessel vasculitis at presentation, the patient received intravenous thrombolysis with recombinant tissue plasminogen activator according to the WAKE-UP protocol (alteplase, total dose 76 mg), with a door-to-needle time of 101 min. The relatively prolonged door-to-treatment interval was attributable to the WAKE-UP protocol, which required MRI confirmation of DWI–FLAIR mismatch to establish eligibility for thrombolysis in the setting of an unknown time of symptom onset. No significant neurological improvement was observed following thrombolytic therapy.

**Figure 3:**
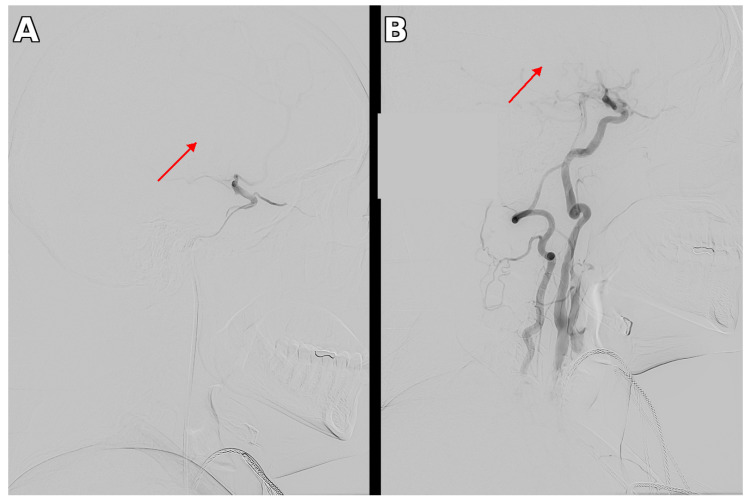
Digital subtraction angiography (DSA). (**A**) DSA confirmed occlusion of the right MCA with minimal residual flow (arrow). (**B**) Post-thrombectomy DSA demonstrated complete recanalization of the right MCA (mTICI grade 3) with restored antegrade flow through the right CCA and ICA (arrow).

Subsequently, the patient was transported to the nearest comprehensive stroke center with endovascular capabilities, located approximately 100 km from her place of residence (door-to-groin time: 200 min). Via right femoral artery access, mechanical thrombectomy (MT) of the right middle cerebral artery was performed first using a thromboaspiration technique, achieving complete recanalization (mTICI grade 3). During the same session, balloon angioplasty of the right common carotid artery and right subclavian artery was performed (Figure 3).

### 3.2. Follow-Up and Outcomes

**Figure 4:**
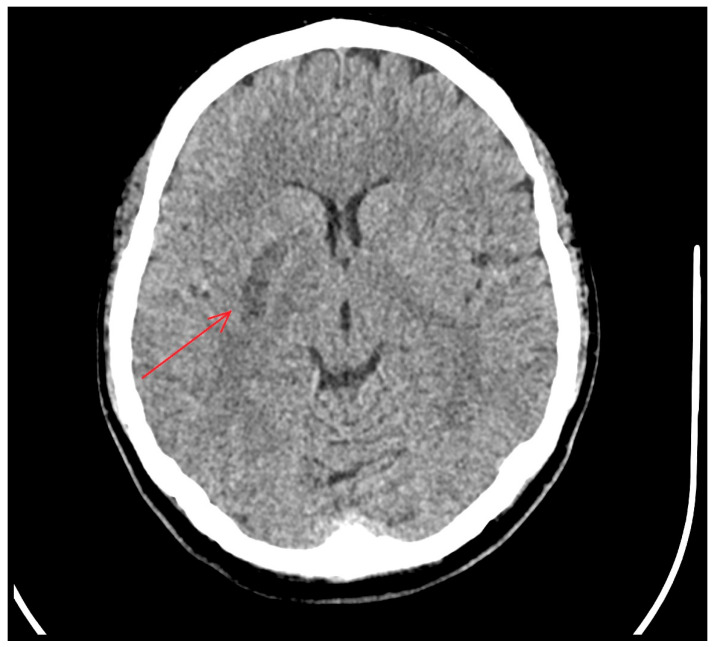
Non-contrast CT scan 24 h after mechanical thrombectomy demonstrated ischemic changes in the right basal ganglia involving the lentiform nucleus (arrow), with no evidence of intracranial hemorrhage.

Following the procedure, the patient’s neurological status improved, with an NIHSS score of 3. A follow-up non-contrast brain CT performed 24 h later showed no intracranial hemorrhage and demonstrated ischemic changes in the right basal ganglia, involving the lentiform nucleus and the head of the caudate nucleus (Figure 4).

Dual antiplatelet therapy with acetylsalicylic acid (ASA) 75 mg/day and clopidogrel 75 mg/day, along with atorvastatin 40 mg/day, was initiated for secondary stroke prevention.

Carotid duplex ultrasonography performed during hospitalization confirmed complete occlusion of the right common carotid artery (CCA) filled with hypoechoic thrombus, with a diameter of 10 mm (compared with 7 mm in the left CCA), and retrograde flow in the right external carotid artery. The left carotid arteries were patent without evidence of stenosis, and the right vertebral artery demonstrated a tortuous course.

Laboratory analysis revealed elevated inflammatory markers, including an erythrocyte sedimentation rate (ESR) of 54 mm/h (reference range 0–15 mm/h), C-reactive protein (CRP) of 24 mg/L (0–10 mg/L), and fibrinogen of 485 mg/dL (200–400 mg/dL). Additional findings included iron deficiency anemia (serum iron 20 µg/dL; reference range 50–170 µg/dL) and the presence of rheumatoid factor (RF).

The patient was evaluated by a rheumatologist. Physical examination at that time revealed a diminished radial pulse on the right side. Blood pressure measurements demonstrated significant inter-arm asymmetry: 117/91 mmHg in the right arm versus 139/100 mmHg in the left arm, with home measurements showing a systolic difference exceeding 50 mmHg. Given the clinical and imaging findings suggestive of Takayasu arteritis, intravenous high-dose methylprednisolone (0.5 g/day) was administered for five consecutive days. The patient’s neurological condition stabilized during hospitalization, with no recurrent ischemic events. She was discharged after 9 days of hospitalization in good general condition, with mild residual left-sided hemiparesis (NIHSS = 2, mRS = 1).

## 4. Follow-Up

**Figure 5:**
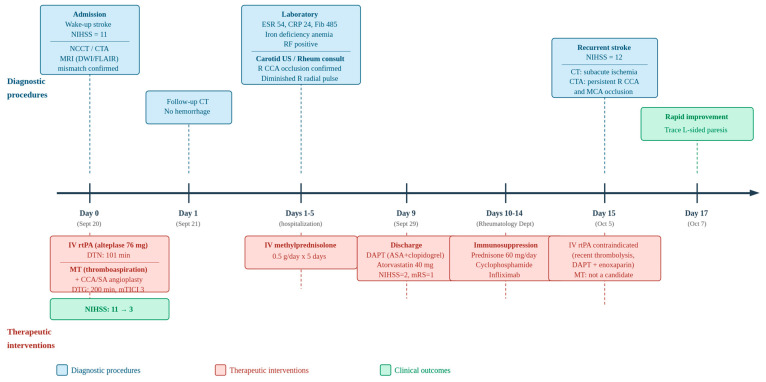
Clinical timeline of diagnostic procedures (above axis) and therapeutic interventions (below axis). Green boxes indicate clinical outcomes. CCA = common carotid artery; CTA = computed tomography angiography; DAPT = dual antiplatelet therapy; DTG = door-to-groin; DTN = door-to-needle; DWI = diffusion-weighted imaging; ESR = erythrocyte sedimentation rate; FLAIR = fluid-attenuated inversion recovery; MCA = middle cerebral artery; mRS = modified Rankin Scale; MT = mechanical thrombectomy; mTICI = modified Treatment in Cerebral Ischemia; NIHSS = National Institutes of Health Stroke Scale; RF = rheumatoid factor; rtPA = recombinant tissue plasminogen activator; SA = subclavian artery; US = ultrasonography.

After discharge from the Neurology Department, she was referred to the Rheumatology Department for ongoing management, where oral corticosteroid therapy with prednisone 60 mg/day was continued, and additional immunosuppressive treatment with cyclophosphamide and infliximab was initiated. A schematic overview of the clinical timeline is presented in Figure 5.

According to the medical documentation provided by the patient, during a subsequent hospitalization at the Rheumatology Department, approximately two weeks after the initial stroke, she experienced a recurrent cerebrovascular event. On admission, the NIHSS score was 12, with left upper extremity plegia, severe left lower extremity paresis, and left-sided hemihypoesthesia. Computed tomography demonstrated subacute ischemic changes in the deep structures of the right cerebral hemisphere. CTA revealed persistent occlusion of the right MCA at the bifurcation with poorly opacified M2 branches suggestive of residual thrombi, complete occlusion of the right CCA along its entire length with a mobile thrombus at the terminal segment, and patent right internal carotid artery with possible segmental wall thickening and 60–70% stenosis in both the extracranial and intracranial segments. Intravenous thrombolysis was contraindicated given the recent thrombolytic treatment and ongoing dual antiplatelet therapy with therapeutic-dose anticoagulation (enoxaparin 0.8 mg twice daily). Following conventional angiography, the patient was not deemed a candidate for mechanical thrombectomy. Notably, her neurological status improved rapidly, with only trace left-sided paresis documented two days after the event.

## 5. Discussion

Ischemic stroke in young adults represents a diagnostic challenge and requires a broad etiological work-up, as uncommon causes are more prevalent in this population compared to older patients. Autoimmune and inflammatory disorders, including large-vessel vasculitides, should be considered particularly in young women presenting with cerebrovascular events, even in the absence of overt systemic symptoms. TA is a chronic granulomatous vasculitis predominantly affecting the aorta and its major branches and may lead to cerebral ischemia through progressive arterial stenosis, occlusion, or thrombus formation.

TA occurs worldwide but shows a higher prevalence in Asian countries, most commonly affecting women between 10 and 40 years of age. Incidence rates vary geographically, from 2.6 per million in Europe and North America to 40 per million in Asia [3,7]. Several studies have reported a prevalence ranging from 4.7 to 33 cases per million in the European population [8,9].

The pathogenesis of this inflammatory disorder remains unclear. Abnormal activation of macrophages and CD4+/CD8+ T cells leads to granulomatous inflammation of large vessels such as the aorta. Matrix metalloproteinase activity contributes to fibrosis and vessel wall thickening, resulting in stenosis or dilatation [10,11].

The clinical presentation of TA is heterogeneous, ranging from an asymptomatic course—especially in the early stages—to general constitutional symptoms and end-organ manifestations [3,12]. TA typically evolves through two phases. The first, systemic phase is characterized by nonspecific symptoms, such as weight loss, fever, myalgia, and arthralgia. In our patient, the reported joint pain previously diagnosed as rheumatoid arthritis may have represented the initial, nonspecific manifestation of TA. In such cases, vascular imaging plays a crucial role in identifying inflammatory changes in the arterial wall and differentiating vasculitis from other causes of large-vessel occlusion, such as arterial dissection or embolic disease. The second phase is defined by vessel stenosis or occlusion, with neurological symptoms including headache, dizziness, blurred vision, syncope, transient ischemic attacks (TIAs), and, as in our patient, stroke [12,13].

Acute ischemia and infarction leading to stroke can result from thrombosis or, more frequently, embolism from affected vessels. Stroke occurs in approximately 10–20% of patients with TA, with about 80% involving the anterior circulation [3,14,15]. However, stroke as the initial manifestation is observed in only 5–8% of cases. The presentation of our patient with acute MCA stroke as the first manifestation of TA was therefore unusual [3,16].

There are currently no universally accepted diagnostic criteria for TA. Existing systems, such as the 1990 American College of Rheumatology (ACR) criteria and the 2022 DCVAS update, were primarily developed for research purposes rather than for routine clinical use [17,18]. Diagnosis relies on clinical assessment supported by vascular imaging, typically magnetic resonance angiography (MRA) or CTA. The 2018 EULAR guidelines provide TA-specific diagnostic and management recommendations [7,19].

In our patient, the differential diagnosis included rheumatoid vasculitis and Lyme-associated vasculopathy, given the pre-existing suspicion of rheumatoid arthritis and concurrent Lyme disease. However, rheumatoid vasculitis predominantly affects small- and medium-sized vessels (e.g., vasa nervorum, cutaneous arterioles) and is not typically associated with large-vessel involvement. Similarly, although neuroborreliosis may rarely cause cerebral vasculitis, it primarily involves small- and medium-sized intracranial vessels. The pattern observed in our patient—inflammatory wall changes in the aortic arch branches including the brachiocephalic trunk, bilateral subclavian arteries, and common carotid artery—is characteristic of large-vessel vasculitis and inconsistent with either rheumatoid or Lyme-associated vasculopathy. Furthermore, clinical findings including a diminished right radial pulse and significant inter-arm blood pressure asymmetry (systolic difference exceeding 50 mmHg on home measurements) fulfilled the ACR criterion of a blood pressure difference greater than 10 mmHg between the arms, providing additional support for the diagnosis of TA.

TA has historically been referred to as “pulseless disease” due to the characteristic absence or diminution of peripheral pulses resulting from large-vessel stenosis. In the acute stroke setting, however, standardized protocols prioritize rapid neuroimaging and time-to-treatment, and a detailed vascular examination including palpation of peripheral pulses and auscultation for bruits over the cervical and supraclavicular regions may not be routinely performed. In our patient, pulse asymmetry and blood pressure discrepancy were documented during rheumatological evaluation but had not been assessed at initial presentation. This case highlights the importance of a thorough vascular physical examination in young stroke patients, as findings such as absent or diminished pulses and arterial bruits may provide early clues to underlying large-vessel vasculitis, even before imaging confirmation. The same principle applies to other large-vessel pathologies presenting as acute stroke, such as aortic dissection.

The primary goal of treatment is to achieve and maintain immunosuppression. Systemic corticosteroids remain the first-line therapy to induce remission, although their long-term use is limited by high relapse rates and adverse effects. Therefore, additional nonsteroidal immunosuppressive agents, such as methotrexate or cyclophosphamide, are often required for sustained disease control [3,7,20]. Following the acute phase, immunosuppressive therapy is fundamental to control vascular inflammation and reduce the risk of recurrent ischemic events. Early collaboration between neurologists, interventional neuroradiologists, and rheumatologists is therefore essential to ensure both acute management and long-term disease control [19,21,22].

Regarding the acute management of ischemic stroke in TA, particularly the use of MT and stenting for large cerebral artery occlusion, only a few cases have been reported in the literature [6,7,13,21,22,23,24,25,26]. Nevertheless, collected case reports and small series suggest that intravenous thrombolysis and MT can be feasible and effective in selected patients with Takayasu arteritis presenting with acute ischemic stroke. In our patient, combined reperfusion therapy resulted in substantial neurological improvement without hemorrhagic complications, supporting the potential role of endovascular treatment when carefully individualized.

During follow-up, while the patient was hospitalized in the Rheumatology Department, a recurrent cerebrovascular event occurred approximately two weeks after the initial stroke, presenting with an NIHSS score of 12. Intravenous thrombolysis was contraindicated due to the recent thrombolytic treatment and ongoing antithrombotic therapy, and the patient was not deemed a candidate for mechanical thrombectomy following conventional angiography. Imaging revealed persistent occlusion of the right CCA, residual thrombi in the right MCA territory, and possible segmental wall thickening of the right internal carotid artery consistent with inflammatory vasculopathy. Despite the severity of the recurrent event, the patient’s neurological status improved rapidly, with only trace left-sided paresis two days later. This recurrent event highlights the high risk of ongoing vascular inflammation in TA and underscores the importance of early immunosuppressive therapy in addition to acute reperfusion strategies.

The present case underscores the importance of prompt reperfusion therapy. The combination of intravenous thrombolysis and MT resulted in significant neurological improvement despite extensive vascular involvement. Reported outcomes in stroke secondary to TA are variable and largely depend on the timing of intervention and the extent of vascular disease. This case demonstrates the technical feasibility and short-term efficacy of MT in the acute treatment of stroke associated with TA, with no procedural complications and substantial immediate neurological recovery. However, the subsequent recurrent cerebrovascular event highlights that successful acute reperfusion does not eliminate the long-term risk of recurrence in the setting of ongoing vascular inflammation. Our experience suggests that early and sustained immunosuppressive therapy is essential to complement acute endovascular strategies and reduce the risk of restenosis and recurrent ischemic events.

## 6. Conclusions

Stroke in young adults requires a comprehensive diagnostic evaluation to identify underlying autoimmune diseases, including large-vessel vasculitis. Although ischemic stroke is a relatively rare initial manifestation of TA, it should be considered in the differential diagnosis of young patients presenting with cerebrovascular symptoms, particularly when large-vessel involvement is identified on imaging.

Diagnosing TA remains challenging due to nonspecific early symptoms, such as musculoskeletal pain, as observed in our patient. A thorough vascular physical examination—including palpation of peripheral pulses and assessment of inter-arm blood pressure differences—may provide early diagnostic clues, even in the acute stroke setting. This case demonstrates that early reperfusion therapy, combining intravenous thrombolysis and mechanical thrombectomy, may achieve immediate neurological improvement even in the presence of large-vessel occlusion; however, the risk of recurrence remains substantial without adequate immunosuppressive control of the underlying vasculitis.

Overall, this report underscores the importance of early recognition of inflammatory arteriopathy in young stroke patients. A detailed vascular physical examination should complement standard acute stroke protocols, as clinical signs such as pulse asymmetry and blood pressure discrepancies may guide early diagnosis. MT in combination with intravenous thrombolysis may be a viable acute therapeutic option in carefully selected cases, but long-term outcomes depend on timely initiation and maintenance of immunosuppressive therapy. A multidisciplinary approach involving neurologists, interventional neuroradiologists, and rheumatologists is essential to optimize both acute neurological outcomes and long-term disease management.

## Data Availability

Data supporting the findings of this study are contained within the article. Further data are not publicly available due to patient privacy and ethical restrictions.

## References

[1] Feigin V.L., Brainin M., Norrving B., Martins S.O., Pandian J., Lindsay P., Grupper M.F., Rautalin I. (2025). World Stroke Organization: Global Stroke Fact Sheet 2025. Int. J. Stroke.

[2] Smajlović D. (2015). Strokes in young adults: Epidemiology and prevention. Vasc. Health Risk Manag..

[3] Memon T., Shekha T.A.M., Acharya P., Nishu R.I., Akhter N. (2022). A Case Report of Takayasu’s Arteritis with Cerebral Infarction As Initial Presentation. Cureus.

[4] Wiszniewska M., Członkowska A. (2008). Rola zapalenia naczyń w etiologii udaru mózgu. Udar. Mózgu..

[5] Szmyrka-Kaczmarek M., Budrewicz S. (2011). Zapalenia naczyń w etiopatogenezie udaru mózgu. Pol. Prz. Neurol..

[6] Tatsuno K., Ueda T., Usuki N., Otsubo H., Araga T., Yoshie T., Takaishi S., Yoshida Y., Ono H. (2021). A Case of Acute Ischemic Stroke Treated with Endovascular Treatment for Tandem Occlusion of the Common Carotid Artery and Internal Carotid Artery Terminal Portion Related to Takayasu Arteritis. J. Neuroendovasc. Ther..

[7] Chirico C., Giorgianni A., Clivio V., Malnati S., Gatta T., Vizzari F.A., Pellegrino C., Fusco M., Piacentino F., Venturini M. (2025). Long-term successfully endovascular treatment of a complicated Takayasu’s arteritis with thrombectomy and stenting: A case report. J. Med. Case Rep..

[8] Sgouropoulou V., Vargiami E., Kyriazi M., Kafterani S., Stabouli S., Tsigaras G., Anastasiou A., Trachana M., Zafeiriou D. (2024). Recurrent Stroke as a Presenting Feature of Takayasu Arteritis in an Adolescent: A Case Report and Literature Review. Prague Med. Rep..

[9] Onen F., Akkoc N. (2017). Epidemiology of Takayasu arteritis. Presse Med..

[10] Owino C., Sirera B., Tarus F., Ganda B., Oduor C., Siika A. (2023). Ischemic stroke at first presentation of Takayasu arteritis in a young African male from Kenya, East Africa: Case report and brief literature review. Clin. Case Rep..

[11] Arnaud L., Haroche J., Mathian A., Gorochov G., Amoura Z. (2011). Pathogenesis of Takayasu’s arteritis: A 2011 update. Autoimmun. Rev..

[12] Oli P., Poudel P., Kc S., Thapa N., Kc A. (2024). Takayasu arteritis presenting as a stroke in young: A case report. Ann. Med. Surg..

[13] Davari P., Sutton P., Jones K.S. (2020). Stroke as the initial presentation of Takayasu’s arteritis: A case report. Radiol. Case Rep..

[14] Kerr G.S., Hallahan C.W., Giordano J., Leavitt R.Y., Fauci A.S., Rottem M., Hoffman G.S. (1994). Takayasu arteritis. Ann. Intern. Med..

[15] Field K., Gharzai L., Bardeloza K., Houghton B. (2017). Takayasu arteritis presenting as embolic stroke. BMJ Case Rep..

[16] Aydin F., Acar B., Uncu N., Başaran Ö., Adalet Yildiz E., Güven A., Çakar N. (2020). Takayasu Arteritis: A Case Presenting with Neurological Symptoms and Proteinuria. Arch. Rheumatol..

[17] Arend W.P., Michel B.A., Bloch D.A., Hunder G.G., Calabrese L.H., Edworthy S.M., Fauci A.S., Leavitt R.Y., Lie J.T., Lightfoot R.W. (1990). The American College of Rheumatology 1990 criteria for the classification of Takayasu arteritis. Arthritis Rheum..

[18] Grayson P.C., Ponte C., Suppiah R., Robson J.C., Gribbons K.B., Judge A., Craven A., Khalid S., Hutchings A., Danda D. (2022). DCVAS Study Group. 2022 American College of Rheumatology/EULAR classification criteria for Takayasu arteritis. Ann. Rheum. Dis..

[19] Hellmich B., Agueda A., Monti S., Buttgereit F., de Boysson H., Brouwer E., Cassie R., Cid M.C., Dasgupta B., Dejaco C. (2020). 2018 Update of the EULAR recommendations for the management of large vessel vasculitis. Ann. Rheum. Dis..

[20] Khealani B.A., Baig S.M. (2002). Takayasu’s arteritis presenting as ischemic stroke—Case report. J. Pak. Med. Assoc..

[21] Mason J.C. (2010). Takayasu arteritis—Advances in diagnosis and management. Nat. Rev. Rheumatol..

[22] Russo R.A.G., Katsicas M.M. (2018). Takayasu Arteritis. Front. Pediatr..

[23] Mangiardi M., Bravi M.C., Pezzella F.R., Ricci L., Anticoli S. (2020). Unsuccessful Endovascular Treatment in a Patient with Stroke Onset of Takayasu Arteritis and Positive Clinical Outcome. Cureus.

[24] Hedna V.S., Patel A., Bidari S., Elder M., Hoh B.L., Yachnis A., Waters M.F. (2012). Takayasu’s arteritis: Is it a reversible disease? Case Report and Literature Review. Surg. Neurol. Int..

[25] Komatina N., Lepić T., Labović B., Stevović T., Petronijević M., Radovinović-Tasić S., Obradović D. (2016). Relapse of Takayasu arteritis as a cause of suicidal poisoning and subsequent major ischemic stroke successfully treated with thrombolytic therapy. Vojnosanit. Pregl..

[26] Ebata T., Uemura J., Yamazaki H., Imai K., Yagita Y. (2017). A Case of Takayasu Arteritis with Acute Bilateral Occlusion of the Internal Carotid Arteries. Brain Nerve.

